# Orphan Designation and Cisplatin/Hyaluronan Complex in an Intracavitary Film for Malignant Mesothelioma

**DOI:** 10.3390/pharmaceutics13030362

**Published:** 2021-03-09

**Authors:** Sabrina Banella, Eride Quarta, Paolo Colombo, Fabio Sonvico, Antonella Pagnoni, Fabrizio Bortolotti, Gaia Colombo

**Affiliations:** 1 Department of Life Sciences and Biotechnology, University of Ferrara, Via Fossato di Mortara 17/19, 44121 Ferrara, Italy; sabrina.banella@unife.it (S.B.); brf@unife.it (F.B.); 2 Food and Drug Department, University of Parma, Parco Area delle Scienze 27A, 43124 Parma, Italy; eride.quarta@studenti.unipr.it (E.Q.); fabio.sonvico@unipr.it (F.S.); 3 PlumeStars srl, c/o Food & Drug Department, Parco Area delle Scienze 27A, 43124 Parma, Italy; paolo.colombo@plumestars.com; 4 Department of Chemical, Pharmaceutical and Agricultural Sciences, University of Ferrara, Via Luigi Borsari 46, 44121 Ferrara, Italy; antonella.pagnoni@unife.it

**Keywords:** cisplatin, sodium hyaluronate, complex, film, intracavitary, intrapleural, mesothelioma, orphan designation

## Abstract

Pleural mesothelioma is a lung diffuse tumor, whose complete resection is unlikely. Consequently, metastases reappear where the primary tumor was removed. This paper illustrates the orphan medicine designation procedure of an intracavitary cisplatin film and related pharmaceutical development aspects requested by the European Medicines Agency (EMA) in its Scientific Advice. Since cisplatin pharmacokinetics from the implanted film in sheep resulted substantially modified compared to intravenous administration, the formation of a cisplatin/hyaluronan complex had been hypothesized. Here, the interaction between sodium hyaluronate (NaHA) and cisplatin (CisPt) was demonstrated. Size exclusion chromatography qualitatively evidenced the complex in the film-forming mixture, only showing the NaHA peak. Atomic absorption spectroscopy of the corresponding fraction revealed platinum, confirming the interaction. Reverse phase HPLC quantified about 5% free cisplatin in the film-forming mixture, indirectly meaning that 95% was complexed. Finally, a study of CisPt release from the film assessed how CisPt/NaHA complex affected drug availability. In water, a medium without chloride ions, there was no release and the film remained intact for 48 h and longer, whereas the placebo film dissolved in 15 min. In 0.9% NaCl medium, the film became more soluble, dissolving within 3–4 h. However, cisplatin release was still controlled by the existing complex in solution until chloride ions displaced it. While the film modified its dissolution with aging, CisPt release remained unaffected (90% released in 48 h).

## 1. Introduction

Diseases affecting fewer than 5 out of 10,000 people are classified as rare in the European Union (EU). The limited prevalence poorly combines with industrial and economic viability of investments, hampering sponsored studies on dedicated medicines for rare diseases. Medicine Agencies, on the other hand, pay great attention to the need of registering medicinal products for rare diseases [1,2]. In fact, the procedures for developing these products by small and medium-size pharmaceutical companies benefit from better use of existing regulatory and process tools toward marketing authorization. For example, an orphan medicinal product, once registered, obtains a market exclusivity of 10 years in the EU and 7 years in the USA. These efforts by regulatory authorities created not only great social attention, but also a growing economic interest for the development of orphan medicines [3,4].

Rare diseases are generally of genetic origin and remedial therapies require medicines that can repair the gene damage. However, their pathological manifestations affect the quality of life and dedicated medicines often containing known drugs, can be proposed [5]. In this case, innovation in the field of drug delivery can tackle the rare disease symptoms without affecting the body with undesirable effects. To this end, drugs can be repurposed via pharmaceutical technological innovations, resulting into new orphan medicines acting by targeting the disease symptoms. Thus, the quality of life of the affected population, waiting for resolutive medical interventions, can be improved.

Mesothelioma is a rare malignancy attacking the mesothelium, i.e., the cells lining pleural and peritoneal cavities and the pericardium. Most cases of malignant mesothelioma are pleural in origin (malignant pleural mesothelioma (MPM)) and related to professional and/or environmental exposure to asbestos fibers. MPM is a very aggressive form of cancer, with a low expectancy of life. The neoplasia appears long time after the inhalation of asbestos fibers [6]. Nowadays, MPM surgical therapy does not completely cure it, since frequent local recurrences or metastases appear in the resected area [7,8].

A novel loco-regional treatment to apply to the mesothelium from where the primary tumor has been removed, can improve the existing therapy, directly attacking the site of recurrences. Our group has developed a sodium hyaluronate (NaHA) film loaded with cisplatin (CisPt) for the treatment of mesothelioma [9,10,11,12]. The film can be stuck to mesothelial surfaces during surgery, determining high and prolonged local cisplatin concentrations at the site where the tumor was removed. To obtain the same drug concentration by intravenous administration, the patient would be exposed to unacceptable toxicity. The expected effect in humans is a long-lasting prevention of local recurrences and an increase in life expectancy.

Cisplatin-loaded films tested on animal models (rat and sheep) revealed significant benefits over conventional treatments as recognized by the European Medicines Agency (EMA) orphan designation report (see public opinion document) [9,11,12,13]. The intrapleural cisplatin film application in an MPM rat model demonstrated the reduction of the inoculated tumor volume and absence of recurrences [9]. A second pharmacokinetics and toxicity study in sheep tested in larger animals the pleural application of the cisplatin/hyaluronan film in order to assess the film manageability in surgical practice, drug release and bioavailability [11]. The physical and mechanical properties of the film enable to cover the pleural surface. In the sheep model, cisplatin film maintained for a long time a high drug concentration at recurrence sites and related lymph nodes. This paired with the prolonged drug release from the film observed in vitro. Moreover, the hepatic and renal toxicity in animals treated with cisplatin films was not significantly different from the control (no treatment), but consistently lower compared to the groups treated with intravenous or intracavitary cisplatin solution [11].

Thanks to these preclinical data, cisplatin/hyaluronan intrapleural film was designated as Orphan Medicinal Product in the European Union on 29 August 2016 (EU/3/16/1719) [13] and then, in 2017, in the USA by the Food and Drug Administration (FDA) [14], with the indication for disease recurrence prevention after surgery in malignant mesothelioma.

The first aim of this paper has been to illustrate the orphan designation procedure of the anticancer cisplatin repurposed as intracavitary film for the prevention of MPM recurrences after primary tumor removal. A drug delivery strategy was applied to construct a biodegradable film for intracavitary application during surgery. At the same time, since the blood level of cisplatin, measured as platin in an ovine model, was unexpectedly high in comparison to intravenous administration of same dose [11], the hypothesis of a complex formation between cisplatin and hyaluronan needed to be verified. In fact, the Protocol Assistance letter by the EMA CHMP (Committee for Medicinal Products for Human Use), received after orphan designation (i.e., the Scientific Advice) for assisting the market authorization activities of the Applicant, asked to distinguish the presence of free and complexed cisplatin. Consequently, the second aim has been to develop a dedicated pharmaceutical study for searching and quantifying the cisplatin complex in order to justify the favorable pharmacokinetics (PK), efficacy and toxicity results previously found in the animal model.

## 2. Materials and Methods

### 2.1. Materials

Cisplatin with purity higher than 99.9% was obtained from Sigma Aldrich (Saint Louis, MO, USA). Sodium hyaluronate (HA ophthalmic, batch A19270, molecular weight 1.33·10^6^ Da) was kindly donated by Fidia Farmaceutici S.p.A. (Abano Terme, Italy). Polyvinyl alcohol was obtained from Nippon-Goshei (PVA; 83,000 Da; Osaka, Japan). Polyethylene glycol 1000 mono-stearate (PEG 1000S; batch 0001459543) was a kind gift of CRODA (Snaith, UK). Polyethylene glycol 200 (PEG 200) and sorbitol were purchased from ACEF (Fiorenzuola d’Arda, Italy). Water for film preparation was Milli-Q ultrapure water (18 MΩ cm, Milli-Q system, Waters Corp., Milford, MA, USA). All other chemicals, reagents and solvents were of analytical grade.

### 2.2. Film-Forming Mixture Preparation and Film Manufacturing

The film used in sheep had square shape (10 cm side) and a thickness of 100–200 µm [11]. In humans, 12 to 15 such films would be required to cover the operated chest surface. Film manufacturing is described in the previous publications [9,10,11,12]. Briefly, the film-forming mixture was prepared as follows: the plasticizing agents, sorbitol (70% *w*/*v*), PEG 1000 stearate and PEG 200, were dissolved in purified water. Then, the film-forming polymer PVA was added; to facilitate its dissolution, the system was heated at 75 °C for two hours. Finally, cisplatin and sodium hyaluronate were added. The mixture was stirred for 24 h until complete polymer hydration. Hyaluronan films were then produced by laminating the viscous mixture on a polyester translucent laminate film (Scotchpak 1220 Backing, 3M Italia, Segrate, Italy) with a variable opening casting knife (BYK Gardner, gap 2 mm; BYK, Geretsried, Germany) in a laminar flow hood. The wet film was then oven-dried at 55 °C for 8 h.

### 2.3. Platinum Quantification by Atomic Absorption Spectroscopy

Cisplatin content in the film was determined based on platinum quantification by atomic absorption spectroscopy. The same technique was used for the assessment of the CisPt/NaHA complex during SEC-HPLC analysis (see Section 2.4). An accurately weighed sample (100 mg for the film) was processed by acid digestion in a microwave oven equipped with SK15 high-pressure rotor (Milestone ETHOS EASY; Milestone, Sorisole, Italy). After cooling, the mixture was transferred to a 50 mL volumetric flask and diluted with Milli-Q ultrapure water (18 MΩ·cm, Milli-Q system; Waters Corp., Milford, MA, USA).

Platinum quantification was carried out on an Analyst 800 atomic absorption spectrometer (Perkin-Elmer, Waltham, MA, USA) equipped with a Zeeman background correction system and an electrothermal atomizer with transversely heated graphite tube. Platinum atomic absorption was measured at 265.9 nm. The method linearity was determined in the range 10–150 µg/L of platinum concentration (y = 0.0005x + 0.0004; R^2^ = 1). Accuracy was assessed by recovery data obtained for spiked real samples, resulting as equal to 80%. A detection limit of 5.66 μg/L was obtained according to the calibration curve method [15].

### 2.4. Chromatographic Analyses

#### 2.4.1. SEC-HPLC

CisPt/NaHA complex was studied by size-exclusion chromatography (SEC-HPLC) coupled with UV-Vis detection (Agilent 1200 series; Agilent, Santa Clara, CA, USA). The stationary phase was a YARRA, SEC-400 column (3 µm, 4.6 × 300 mm; Phenomenex, Torrance, CA, USA). The eluent was an aqueous solution of 50 mM KH_2_PO_4_ and 100 mM Na_2_SO_4_, whose pH was adjusted to 6.8 ± 0.1 with 1 M NaOH. The detection wavelength was set at 210 nm. Isocratic elution was carried out at flow rate of 0.5 mL/min at room temperature; the injection volume was 10 µL. In these conditions, the retention times of NaHA and CisPt were 3.7 and 8.5 min, respectively. The method linearity was confirmed in the 5–200 µg/mL NaHA concentration range (y = 10.728x − 1.2819; R^2^ = 1.0000).

#### 2.4.2. Ion Pair RP-HPLC

Cisplatin in the film-forming mixture was quantified by ion pair reverse phase high performance liquid chromatography (ion pair RP-HPLC) coupled with UV-Vis detection, according to the European Pharmacopoeia method (Ph. Eur. 8.0 Cisplatin monograph: 01/2009:0599 corrected 7.0) with a minimal change in the stationary phase used. The stationary phase was a Zorbax Eclipse XDB-C8 column (5 µm, 4.6 × 150 mm, base-deactivated; Agilent, Santa Clara, CA, USA). Isocratic elution was carried out at room temperature with an aqueous solution of 5 mM sodium 1-octanesulfonate, 5 mM tetrabutylammonium hydrogen sulfate and 20 mM KH_2_PO_4_, adjusted to pH 5.9 ± 0.1 with 1 M NaOH. The flow rate was 0.6 mL/min and the injection volume was 10 µL. The detection wavelength was 210 nm. In these conditions, the retention times of NaHA and CisPt were 1.9 and 2.8 min, respectively. The method linearity was confirmed in the 2–50 µg/mL cisplatin concentration range (y = 14.775x + 2.9054; R^2^ = 0.9999).

#### 2.4.3. RP-HPLC

The simultaneous quantification of NaHA and CisPt in the film-forming mixture and in samples from the release experiments, was performed in different reverse phase chromatographic conditions. The column used was a Synergi Polar-RP (4 µm, 4.6 × 150 mm; Phenomenex, Torrance, CA, USA). Isocratic elution at room temperature was carried out with 25 mM KH_2_PO_4_, adjusted to pH 5.8 ± 0.1 with 1 M KOH. The detection wavelength was 210 nm. The flow rate was 0.6 mL/min and the injection volume 10 µL. In these conditions the retention times of NaHA and CisPt were 1.5 and 3.4 min, respectively. The method linearity was confirmed in the 1.2–46.4 µg/mL cisplatin concentration range (y = 16.065x + 0.4436; R^2^ = 1.0000) and in the 50–250 µg/mL NaHA concentration range (y = 1.616x − 3.1234; R^2^ = 0.9996).

### 2.5. Preparation of Stock, Standard Solutions and Samples of Film-Forming Mixture

Cisplatin stock solution was prepared in 0.9% (*w*/*v*) NaCl at about 0.5 mg/mL concentration. Then, the stock was diluted with mobile phase to final CisPt concentrations in the 25–50 µg/mL range.

Sodium hyaluronate standard solution was prepared by dissolving the polymer powder in mobile phase to a final concentration in the 0.25–1 mg/mL range. Before injection, the NaHA solution was centrifuged for 10 min at 8000 rpm (Beckman Microfuge^®^ 18; Beckman Coulter, Cassina de’ Pecchi, Italy).

For the film-forming mixture assay, an accurately weighed mass of mixture was diluted in order to have a final concentration between 0.5–1 mg/mL for NaHA and between 14–28 µg/mL for CisPt. The samples were centrifuged before analysis (10 min, 8000 rpm).

### 2.6. Cisplatin Release Rate from Films

For determining the release of cisplatin from films, rectangular samples (1 × 7 cm) containing 1% (*w*/*w*) CisPt, were cut, weighed and immersed in glass vials filled with 20 mL of pre-warmed isotonic 0.9% NaCl solution at pH 7.0 ± 0.1. The vials were placed in a water thermostatic bath at 37 °C under magnetic stirring. Samples were withdrawn at specified time points and the corresponding volume was replaced with fresh medium. After centrifugation, the concentrations of NaHA and CisPt in samples were determined by RP-HPLC (see Section 2.4.3).

### 2.7. Statistical Analysis

Data are expressed mean ± standard deviation (SD). They were compared by applying an unpaired two tailed Student’s *t*-test. *p* < 0.05 was considered to indicate statistical significance.

## 3. Results and Discussion

Results are divided in two main sections. First, a concise and accurate presentation of the orphan medicine designation procedure is described. In the second, the experimental results devoted to the CisPt/NaHA complex assessment and its effect on drug release rate, as well as the experimental conclusions that can be drawn, are reported.

### 3.1. EMA Orphan Medicine Application Procedure

EMA encourages sponsors to request a pre-submission meeting with the orphan committee rapporteurs prior to filing the application for orphan medicine designation. The assistance is free of charge and mostly held via teleconference. During the pre-submission meeting, EMA will also offer a quality check of two key aspects, i.e., the application’s draft submission data and the attached scientific document.

The essential steps of the scientific part of the application provide the requested information listed according to five sections, the first of which is the exhaustive description of the disease or rare condition. Thus, the etiology, pathophysiological, histopathological, clinical characteristics, symptoms and diagnosis are described. Pleural mesothelioma is a rare malignancy arising from the mesothelium. The risk of developing mesothelioma depends on the exposure to the asbestos mineral fibers. Inhaled asbestos or other carcinogenic fibers induce cytotoxicity, DNA damage, frustrated phagocytosis and chronic inflammation. Typical presentation is either with chest pain and/or dyspnea. A combination of accurate exposure history, along with diagnostic radiology and pathology, is essential to make a correct diagnosis.

The proposed orphan indication of this cisplatin intracavitary film has been the “Management of residual disease after malignant pleural mesothelioma surgery to prevent loco-regional recurrences”.

The successive section describes the medical plausibility of the proposed medicinal product. Since in many cases, at the time of designation, little or no clinical experience is available, it is important that the relevance of in vitro and in vivo preclinical models presented in the application are discussed in the context of the condition, including the active substance description, pharmacological class and mode of action. Cisplatin is a metal platinum coordination compound approved since 1978 for the treatment by intravenous injection of many hematological and solid tumor malignancies, including malignant mesothelioma [16]. Hyaluronic acid is an endogenous and biocompatible polysaccharide polymer. Cisplatin/hyaluronan film is the novel cisplatin delivery system presented as an adhesive and flexible film [9,10,11,12]. Planar geometry of the dosage form is rational for the deposition or for coating the pleural surface after surgery. The film has to be stuck by gentle pressure on the chest area at risk of recurrence, for the loco-regional therapy. The CisPt/NaHA film is adapted to the application site, allowing bending, folding, cutting and any deformation necessary to cover the pleural surface in a surgical setting. The aim is to inhibit the recurrence in the area where the tumor has been removed, by maintaining the biological interface in contact with the anticancer drug. The efficacy of cisplatin in the film was assessed by means of in vitro studies on A549 NSCLC cell line, whose proliferation was significantly inhibited [10].

Previous studies by other groups described intracavitary therapeutic approaches using solutions of cytotoxic drugs. In these cases, the major issue was the relatively short drug exposure of local tissues, because of exudate dilution, rapid absorption and systemic distribution [17]. On the contrary, in the animal studies with the intracavitary cisplatin/hyaluronan films, prolonged plasma levels of the cytotoxic drug in comparison with intravenous and intracavitary cisplatin solution, were observed [9,11]. Moreover, reduction in tumor recurrences was observed, suggesting that the in vitro prolonged release of cisplatin was present also in vivo. The cisplatin pharmacokinetics supported by the polymeric film, enhanced the local and systemic exposition of the tumor, apparently without increasing side effects [9]. In summary, the in situ prolonged release of cisplatin from the hyaluronan film on mesothelium in a rat tumor model, sustained the anticancer activity and reduced the toxic effects.

Then, the scientific document asks for the justification of the life-threatening or debilitating nature of the condition. Mesothelioma is a very aggressive form of cancer, difficult to diagnose and highly resistant to treatment. Full recovery is extremely rare [18]. MPM is characterized by a peculiar growth extending along the pleural surface with a diffuse and non-spherical pattern [19]. Early diagnosis is so rare that mesothelioma treatment is usually unable to provide a complete cure. Even in early-stage disease, the prognosis remains grim with an overall median survival time of 9–12 months [20,21].

Then, an important section of the application was devoted to the number of people affected by the orphan disease, presented as disease prevalence in the European Union, citing the reference documentation and giving information on the database of rare diseases examined. At the time of designation, malignant mesothelioma affected less than 1 in 10,000 people in the EU. This was equivalent to a total of fewer than 51,000 people, well below the ceiling for orphan designation.

The existing methods for diagnosis, prevention or treatment of the condition have to be illustrated as well. If this is the case, the non-satisfaction of existing methods has to be justified, in order to introduce the significant benefit of the proposed medicine. In detail, the need to improve the chemotherapy efficacy and, particularly, the lack of treatments to reduce local recurrence is evident from recent studies [22,23]. The implant film for intracavitary application, composed of hyaluronan loaded with cisplatin to be used after tumor resection, is presented as the medicinal product addressing this need. Innovation in loco-regional drug administration is the strategy to increase the effectiveness of chemotherapy and to reduce toxic effects of cisplatin in MPM patients undergoing chemotherapy. In summary, the Applicant, at the time of designation, assumes that the new medicinal product of cisplatin releasing the dose on the pleura should bring a significant benefit compared to the existing authorized products and treatments.

Preclinical data supported the benefit provided by the activity of cisplatin intrapleural administration as hyaluronan film for the treatment of MPM. In the already mentioned orthotopic rat model of MPM, intracavitary cisplatin/hyaluronan films resulted more effective than cisplatin solution in reducing tumor recurrences. Plasma concentrations of cisplatin on postoperative days and at autopsy were six-fold higher in the animals treated with cisplatin/hyaluronan films as compared to animals receiving the cisplatin solution. No significant systemic toxicity was observed [9].

Later, in a healthy sheep model, i.e., without inoculating tumor cells, left pneumonectomy was carried out [11]. Thereafter, the intravenous (IV) cisplatin, intracavitary (IC) cisplatin solution and cisplatin/hyaluronan film treatments were performed [11]. Pharmacokinetics parameters of cisplatin with the hyaluronan films showed a delayed T_max_ and a higher area under the curve (AUC_0-t_), compared to the IV and IC administration of the same dose of cisplatin in solution. Locally, the tissues directly in contact with the film, such as the pleural surface or the pericardium, showed a concentration of cisplatin at autopsy significantly higher than the levels obtained using the cisplatin solution administered intravenously or intracavitarily. These results support the claim that the medicinal product CisPt/NaHA, proposed for intrapleural implant, is providing high cisplatin concentrations in those tissues that have the highest likeliness of residual neoplastic cells, accountable for the tumor local recurrence after surgery. Other tumors may benefit from this loco-regional chemotherapy, such as peritoneal mesothelioma, gynecological tumors (ovarian, cervical) or bladder cancer [24,25,26,27]. Moreover, other anticancer drugs could be loaded in the film composition, but in the absence of the formation of a complex with hyaluronan, the film would work only as a polymeric delivery system.

Afterwards, the EMA application requires the description of the development stage of the product, illustrating in a summary the non-clinical aspects (composition, manufacturing, drug release and quality characteristics), the preclinical Proof-of-concept in relevant animal models and the pharmacokinetics and toxicity studies in sheep, concluding with the indication of planned clinical studies.

To finish, details of current regulatory status and marketing history in the EU and non-EU countries and sponsor’s position conclude the application, together with the cited references. The document is sent to the EMA Committee for Orphan Medicinal Products (COMP), which after discussion, frequently in presence of the sponsor, either approves the orphan designation or not. After approval, since the application is filed in a common format between the EMA and FDA, a version of the application in the format required by FDA is sent to the American Agency for the concomitant approval.

A valuable conclusive step is that, upon receiving the approval, the sponsor can apply for Scientific Advice (for orphan medicines it is named Protocol Assistance) to obtain the Agency support for the pharmaceutical, preclinical and clinical phases required for the marketing authorization dossier. The complete procedure is free of charge for small and medium enterprises (SME) and for Universities.

### 3.2. Cisplatin/Hyaluronan Film and CisPt/NaHA Complex Formation Assessment

**Figure 1:**
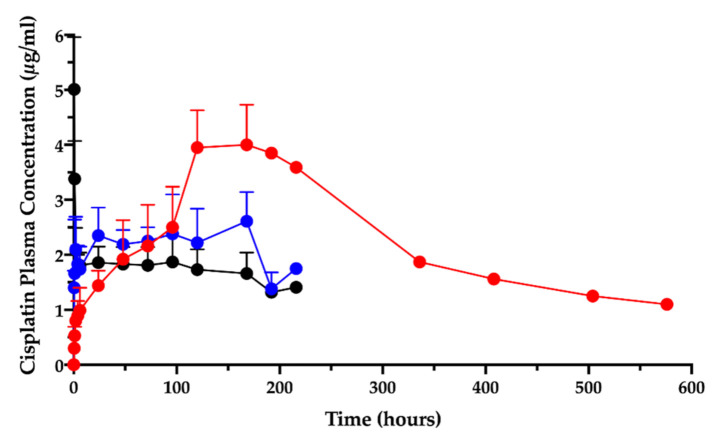
Cisplatin plasma concentration vs. time after intravenous (black) and intracavitary administrations: cisplatin solution (blue), cisplatin/hyaluronan film (red). Data are re-elaborated from Ampollini et al. [11] and expressed as mean ± SD (*n* = 5). One animal in the cisplatin/hyaluronan film group was followed beyond the 9th day post-surgery.

In the animal studies submitted for the orphan designation procedure, in which the cisplatin/hyaluronan film was applied intracavitarily on the operated pleural surface, it was discovered that the intracavitary local absorption provided high local drug content in the mesothelium surfaces [9,11]. Moreover, the PK study after film application in the ovine model revealed that the cisplatin level in plasma, measured as elemental platinum, reflected the drug slow absorption; the AUC_0-t_ increased by more than three times compared to the cisplatin solution given intravenously or intracavitarily (Figure 1) [11].

Surprisingly, the peak of absorption (C_max_) was much higher than the same dose given as intracavitary solution and close enough to the C_max_ of the intravenous injection. Moreover, the film delayed the time-to-peak (T_max_) compared to the systemic administration by injection.

The complexation of cisplatin by hyaluronan was postulated to explain this different kinetics between cisplatin/hyaluronan film and intravenous or intracavitary cisplatin solution. As a consequence, in the EMA Protocol Assistance letter, the Agency required to assess the presence of a CisPt/NaHA complex in the intracavitary film, likely formed during the film preparation, as well as in plasma. The literature reports the formation of a complex between cisplatin and hyaluronan as result of a ligand exchange reaction of cisplatin’s chlorides by the hyaluronan carboxylate groups [28,29]. Platinum is a transition metal element, thus can form coordination compounds (complexes or complex ions) with ligands donating one or more electron pairs to the central metal. Pt^2+^ acts as an electron acceptor (Lewis acid) and the ligand/s as electron donor (Lewis base). The complexation of cisplatin by the polycarboxylated hyaluronan has been exploited to enhance CisPt antitumor activity while decreasing its toxicity. Indeed, Cai and co-authors showed that the intravenous CisPt/NaHA conjugate increased plasma AUC by 2.7 folds compared to a simple cisplatin solution, with a lower C_max_ value [29]. Therefore, the pharmaceutical section of this paper demonstrates the presence of a complex between cisplatin and hyaluronan in the intracavitary film.

#### 3.2.1. Size-Exclusion Chromatography (SEC) Analysis

The surgical film, loading a specific and accurate amount of cisplatin to be targeted to the chest cavity, was manufactured according to Sonvico et al. [10]. This film contains cisplatin combined with the naturally occurring sodium hyaluronate polymer, a ligand for CD44 receptor [30,31]. Thus, sodium hyaluronate acts both as a biocompatible, non toxic and biodegradable polymer for cisplatin delivery and targeting moiety for CD44. The NaHA polymer used here for film construction had an average molecular weight (MW) around 1.33·10^6^ Da, i.e., much higher than the polymer used for the CisPt/NaHA conjugate reported by previous authors [28,29]. The polymer MW impacts the film’s physical structure and its mechanical properties. As the film is intended for intracavitary application, suitable mechanical resistance, tensile strength and flexibility are required.

**Figure 2:**
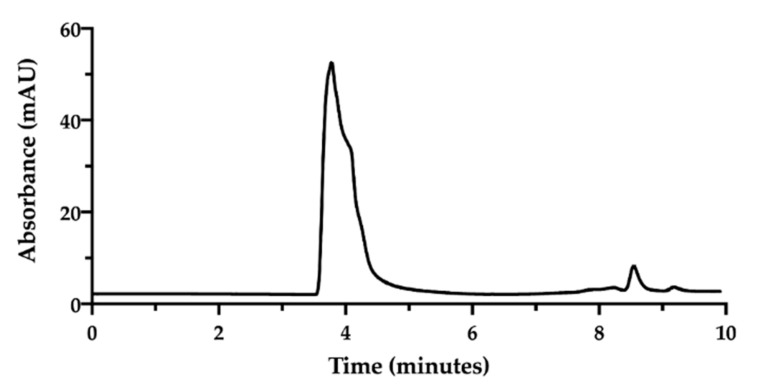
SEC-HPLC chromatogram of sodium hyaluronate solution (1.0 mg/mL) in water.

In order to assess the complex between CisPt and NaHA, we decided to exploit their great difference in molecular weight [32]. An HPLC method was developed using a Size Exclusion Chromatography (SEC) column. In SEC conditions, an NaHA aqueous solution (standard) produced a chromatogram exhibiting the hyaluronan peak at 3.7 min as retention time. Then, between 8.0 and 9.0 min, small peaks appeared due to the elution of low molecular weight species in the solvent (Figure 2).

**Figure 3:**
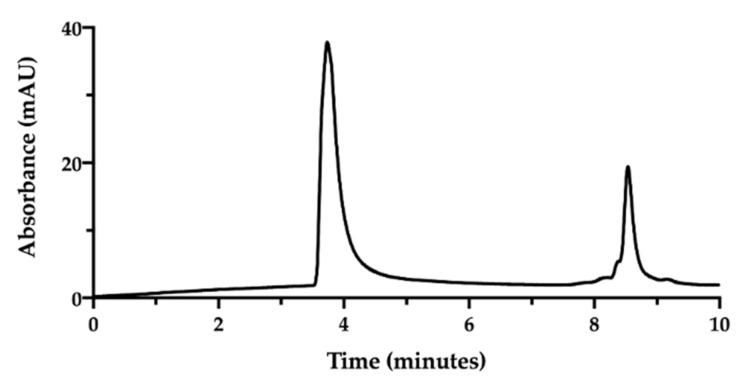
SEC-HPLC chromatogram of NaHA (0.5 mg/mL) and CisPt (25 µg/mL) mixture in water injected immediately after preparation.

Aiming to pursue the chromatographic separation between NaHA and CisPt, a mixture 1:1 of NaHA solution (1.0 mg/mL) and CisPt solution (50 µg/mL), was injected immediately after mixing (Figure 3).

**Figure 4:**
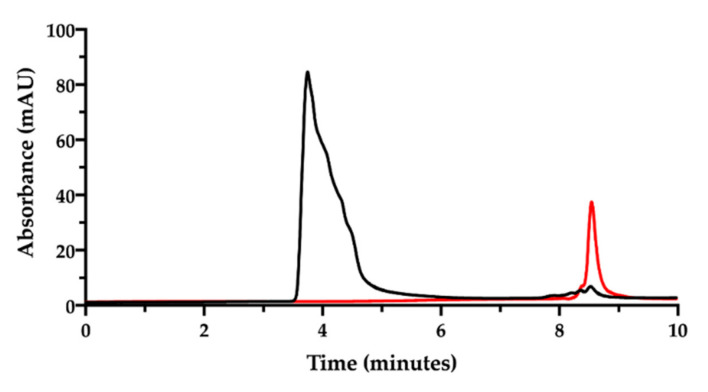
SEC-HPLC chromatogram of the film-forming mixture (NaHA 1 mg/mL; CisPt 28 µg/mL) (black). The chromatogram of CisPt standard solution (50 µg/mL) (red) is superimposed.

In the applied SEC-HPLC conditions, the NaHA peak was confirmed at 3.7 min, while the CisPt peak appeared at 8.5 min, completely overlapping the peaks of the low molecular size species (Figure 3). Consequently, the separation obtained by SEC-HPLC allowed the identification of the two components of the complex. Concerning the quantification of cisplatin in this mixture, the co-elution with the other low molecular weight substances in solution diminished the specificity of the assay. However, in this mixture, the CisPt retention time was 8.5 min, both in the presence and absence of NaHA. In Figure 3, the height of the CisPt peak was about one half as compared to the peak obtained by injecting the 50 µg/mL cisplatin standard solution by itself (see next Figure 4). This indicates that in the injected CisPt/NaHA mixture, no complex was formed between cisplatin and hyaluronan in the short time from preparation to analysis.

Now, in order to simultaneously determine the content of NaHA and CisPt and their interaction, the film-forming mixture was prepared according to Sonvico and co-workers [9,10,11,12]. This preparation containing the two substances, was kept at room temperature for one week, then analyzed after dilution with mobile phase at a final NaHA concentration of 1.0 mg/mL. The expected CisPt concentration in the diluted solution was 28 µg/mL. The chromatogram corresponding to this preparation is shown in Figure 4.

In the chromatogram of the film-forming mixture (black trace in Figure 4), no evident peak assignable to CisPt was recorded at 8.5 min, but only the NaHA peak and the small peaks due to the eluent. The NaHA peak’s height and width in the film-forming mixture were different from the analysis of the NaHA standard solution at the same concentration (see Figure 2). It was verified that the greater area under the NaHA peak in the film-forming mixture did not come from the contribution of the other polymeric species present, namely PVA (chromatogram available in the Supplementary Material, Figure S1). The difference in peak shape and height could be then assigned to a change of molecular size and UV absorbance of the hyaluronan polymer, likely due to the interaction with CisPt. However, it should also be considered that the concentration of the sample from the film-forming mixture was out of the NaHA linearity range (5–200 µg/mL). Figure 4 was drawn superposing the chromatogram of the 50 µg/mL CisPt standard solution (red trace), whose concentration was twice the CisPt concentration expected in the film-forming mixture. Therefore, the lack of CisPt peak in the film-forming mixture’s chromatographic trace can be indirect evidence of drug complexation by the hyaluronan polymer.

This result opened to the hypothesis that CisPt, coordinated by NaHA to form the complex, was eluted beneath the peak assigned to hyaluronan. To demonstrate the defensibility of this assumption, some runs of the same film-forming mixture were carried out to collect the eluate from 1.0 to 4.0 min. This is the time interval comprising the retention time of the NaHA peak. The collected eluate was freeze-dried and analyzed by atomic absorption spectroscopy to verify the presence of platinum. This analysis confirmed that there was platinum in the liquid eluted across the retention time of sodium hyaluronate, likely complexed with the polymer. However, the difficulty in quantitatively collecting the sample only allowed qualitative identification of the metal presence, whereas its quantification was not accurate enough (only 50% of the expected cisplatin was recovered).

#### 3.2.2. Ion Pair Reverse Phase HPLC Analysis

Having verified the unsuitability of SEC-HPLC technique for the quantitative analysis of low molecular weight substances, the evoked formation of the CisPt/NaHA complex in both the film-forming mixture and dry film was studied by ion pair RP-HPLC. In fact, this technique is suitable for the separation of organic ions and partly ionized organic species [33]. Preliminarily, we focused on the hydrolysis of cisplatin in water. In fact, as also reported in the European Pharmacopoeia (Cisplatin monograph), the quantification of cisplatin in a product relies on the sum of the areas of both CisPt peak and platinum aquo complex peak, when present. In this regard, Hindmarsh reported that “In the earliest biological experiments with cisplatin it was noted that there was an initial insensitivity to the drug effect, lasting about two hours. It was suggested that cisplatin was over time converted to the actual ‘active’ form of drug. Studies on the aqueous chemistry of cisplatin can help to explain this activation. In aqueous solution the labile chloride ligands are displaced in a stepwise manner by water molecules as shown in the following equations (Equations (1) and (2)):

(1) $$\mathrm{cis}-[{\mathrm{PtCl}}_{2}({\mathrm{NH}}_{3}{)}_{2}]\;\overset{k_{1}}{\underset{k_{-1}}{⇌}}\;\mathrm{cis}-[\mathrm{PtCl}({\mathrm{NH}}_{3}{)}_{2}({\mathrm{OH}}_{2}){]}^{+}\;+\;{\mathrm{Cl}}^{-},$$

(2) $$\mathrm{cis}-[\mathrm{PtCl}({\mathrm{NH}}_{3}{)}_{2}({\mathrm{OH}}_{2}){]}^{+}\;\overset{k_{2}}{\underset{k_{-2}}{⇌}}\;\mathrm{cis}-[\mathrm{Pt}({\mathrm{NH}}_{3}{)}_{2}({\mathrm{OH}}_{2}{)}_{2}]2^{+}\;+\;{\mathrm{Cl}}^{-}\;$$

As water is a much better leaving group than either chloride or hydroxide, the chloroaqua species will be the most likely reactive form of cisplatin in vivo and hydrolysis is the rate-limiting step in the reaction of cisplatin with biomolecules [34].

For this purpose, a stock solution of cisplatin in water (0.5 mg/mL) was prepared for immediate analysis. Then, this same solution was left under stirring for 18 h at room temperature. In both cases, the stock was diluted to 50 µg/mL CisPt with mobile phase prior to injection.

**Figure 5:**
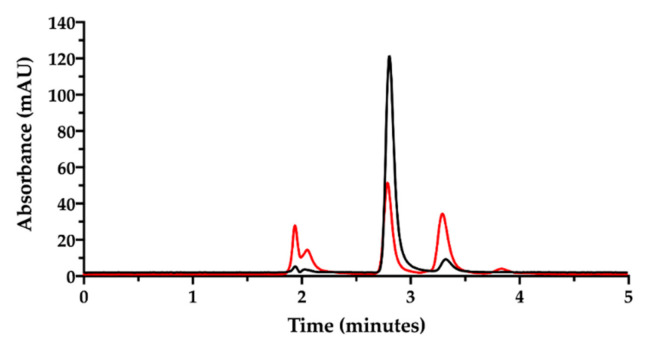
Ion pair RP-HPLC chromatogram of the cisplatin stock solution in water diluted to 50 µg/mL immediately after preparation (black) and after 18 h storage at room temperature (red).

In the chromatogram of Figure 5, the black trace shows the freshly prepared cisplatin solution in water with the CisPt peak at 2.8 min. The second peak at 3.2 min was attributed to the “aquo complex”, according to the European Pharmacopoeia that identifies the “aquo complex” as the peak having a relative retention of about 1.2 with reference to cisplatin. The European Pharmacopoeia does not specify whether this aquo complex is the monoaqua- or diaquacisplatin [35]. Conversely, the red trace in Figure 5 represents the same solution analyzed after being stored for 18 h at room temperature. A reduction in cisplatin peak (2.8 min), an increase in the aquo complex peak at 3.2 min and the appearance of a third little peak at 3.8 min (possibly attributed to diaquacisplatin) were observed. The peaks around 2.0 min derive from the ions in the eluent, which increased during solution storage as chloride ions were displaced by water to form cisplatin aquo complexes. The cisplatin hydrolysis kinetics in water with formation of free chloride has been accurately investigated by Hann and co-workers, also considering the effect of the addition of chloride ions to water [35]. In particular, they observed that when water contained chlorides, hydrolysis occurred to a lesser extent and an equilibrium between cisplatin and its mono- and diaqua- derivatives was reached in 48 h. This was not the case in pure water, where hydrolysis was almost complete in 48 h and the aquo-species were further transformed into unknown platinum compounds. The effect of chloride ions on CisPt hydrolysis was also observed by Karbownik et al., who reported greater stability of cisplatin for injection in physiological solution containing 0.9% NaCl [36].

**Figure 6:**
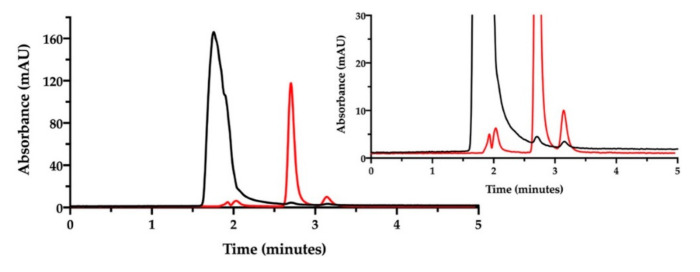
Ion pair RP-HPLC chromatogram of the film-forming mixture diluted in water (black). The chromatogram of cisplatin stock solution in water diluted to 50 µg/mL is superimposed (red). The insert zooms in for a close-up of the eluted peaks.

Then, the ion pair RP-HPLC technique was applied to assess the presence of free and complexed/coordinated cisplatin in the film-forming mixture, i.e., the composition that forms the intracavitary film upon layering and drying. The mixture was analyzed after dilution with water. Under these chromatographic conditions (Figure 6), the retention times were 1.9 min for NaHA and 2.8 min for CisPt, respectively.

#### 3.2.3. Reverse Phase HPLC Analysis

At this point, we recognized that the ion pair RP-HPLC method suffered in specificity for NaHA because the polymer was eluted too close to the solvent front. Moreover, ion pair chromatography can be problematic due to longer column equilibrium times and the higher number of factors that control separation compared to conventional RP-HPLC, often leading to variations in the analyte retention time [37]. Recently, Wan-Hsin Chang et al. proposed for NaHA quantification a “non-conventional chromatography”, meaning that a reverse phase column is used without ion pair and with minimum organic solvent concentration in the mobile phase [38]. In their conditions, sodium hyaluronate was eluted sooner than the solvent front, better separated from salts and low molecular weight compounds.

**Figure 7:**
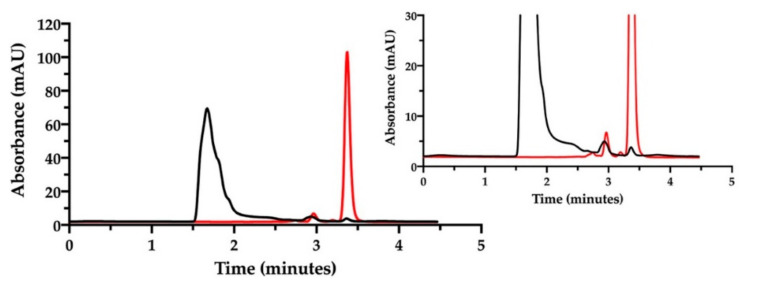
RP-HPLC chromatogram of the film-forming mixture diluted in water (black). The chromatogram of cisplatin stock solution in saline diluted to 46.4 µg/mL is superimposed (red). The insert zooms in for a close-up of the eluted peaks.

We decided to change the stationary phase, choosing a column that was more selective for polar and aromatic compounds, to improve the simultaneous quantification of NaHA and free CisPt in the film-forming mixture. The same investigation done by ion pair chromatography was thus repeated on a batch of film-forming mixture, properly diluted (Figure 7).

Indeed, with this column, NaHA was eluted at 1.5 min, better separated from the solvent front peaks, and cisplatin at 3.4 min. As found with the previous ion pair method, a very low peak of free CisPt was detected in the mixture, corresponding approximately to 5% of the expected CisPt concentration. Hence, 95% of “apparently disappeared” cisplatin was considered to be complexed and co-eluted with hyaluronan. Furthermore, this method enabled the quantification of hyaluronan in the film-forming mixture. All together, these results provide the evidence of complex formation between cisplatin and hyaluronan already in the film-forming mixture, i.e., prior to intracavitary film layering.

#### 3.2.4. In Vitro Cisplatin Release from Film

The improved antitumor activity in rats, the low systemic toxicity and the unexpected pharmacokinetics profile in sheep [9,11] evoked an important biopharmaceutical role of the molecular species CisPt/NaHA complex. Consequently, the understanding of the absorption of cisplatin from the film implanted in the thoracic cavity requires an in vitro release study. The polymeric film loaded with CisPt is a drug delivery system to be intracavitarily implanted. Apart from the considerations on absorption and elimination of cisplatin from the film in the ovine model [11], at this point in the current study the complex presence has been verified only in the film-forming mixture. The actual film is obtained upon layering and drying of such a mixture, raising the question of how this process affects the film rehydration in the presence of the complex. In addition, film dissolution in vivo takes place in the presence of chloride ions that in human plasma are quantified as 103 mM [39]. As said, chloride ions are good ligands for platinum; when available in the release medium, they may displace the platinum ion from the complex competing with the NaHA carboxylate groups in coordinating the metal [40].

**Figure 8:**
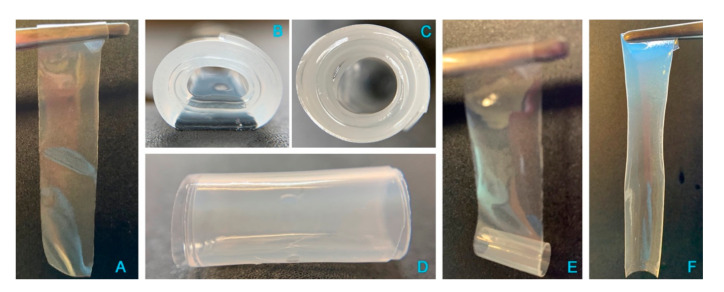
Photographs of the film before and after immersion in water: (**A**) dry film prior to immersion in water; (**B**–**D**) different views of the rolled-up wet film with multilayer structure after 80 h immersion in water at 37 °C; (**E**,**F**) unrolled film cylinder and return to flat geometry.

Preliminarily, the release of cisplatin from a freshly manufactured film was tested in vitro in purified water at 37 °C, i.e., excluding the variable of chloride ions in the medium. Unexpectedly, shortly after being immersed in the aqueous medium, the film changed its morphology (Figure 8); in particular, due to hydration and swelling of its polymeric components, the flat and thin rectangular film (Figure 8, panel A) rolled up on itself to form a cylindrical shape, made of several superimposed layers (Figure 8B–D). The amount of drug released from the system was about 14% of the loaded CisPt in half an hour; then, the following sampling times evidenced a progressive decrease in CisPt concentration in the dissolution medium (data not shown), which was attributed to CisPt hydrolysis. In particular, at 8 h the residual CisPt concentration in solution was 15% of the initial value measured at 30 min. The degradation by hydrolysis followed first order kinetics [y (M) = 2·10^−5^ $e^{{-7\cdot 10}^{-5}x\;(\sec)}$; R^2^ = 0.9988], in line with the trend observed by Hann [35]. However, CisPt degradation was faster in our experiment due to the higher temperature (37 °C vs. 25 °C in Hann’s study). When the experiment was ended at 80 h, no more CisPt was in the solution. Surprisingly, the rolled-up film, more hydrated but minimally swollen, was still present in the release medium at 80 h. Taken out of the medium gently with a pair of tweezers, it unrolled easily (Figure 8E) to restore the original rectangular flat geometry of the film sample cut for the test (Figure 8F). The quantification of only about 32% of hyaluronan in solution at 80 h matched with the “persistence” of the film in water. This could be caused by non-released coordinated platinum, crosslinking adjacent hyaluronan polymer chains. To support this hypothesis, a placebo film without CisPt was immersed in water at 37 °C. In 15 min, the placebo film was completely dissolved, confirming the crosslinking effect of the CisPt/NaHA interaction.

In order to verify the effect on cisplatin release rate by chloride ions, the dissolution test on a freshly manufactured film (1-day-old) was repeated in NaCl 0.9% at 37 °C. Such a test could shed light also on the film dissolution. Both CisPt and NaHA released amounts were determined by RP-HPLC with the Synergi Polar-RP column.

**Figure 9:**
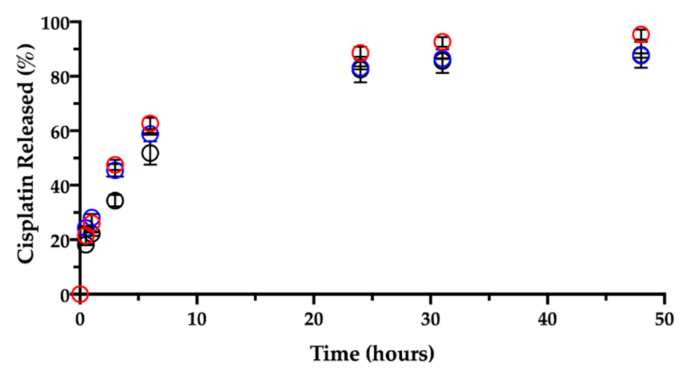
Release of cisplatin in 0.9% NaCl at 37 °C from films aged: 1 day (black), 3 months (red) and 7 months (blue). Data are expressed as mean ± SD (*n* = 3).

Like in water, the contact and immersion in saline solution caused the film to roll up in a few minutes. About 20% (18.2 ± 0.2%) of the CisPt in the film was dissolved in 30 min, determining a burst release (Figure 9).

Ligand exchange and consequent CisPt release were enhanced by the presence of chloride ions as compared to the behavior in water. Indeed, the CisPt release profile continued rather linearly up to six hours. Afterwards, the release rate slowed down, reaching a plateau at 48 h, when almost 90% (87.9 ± 4.8%) of CisPt was found in solution. Hence, while in water the initial release was followed by CisPt hydrolysis, the release profile in saline was almost complete in 2 days.

**Figure 10:**
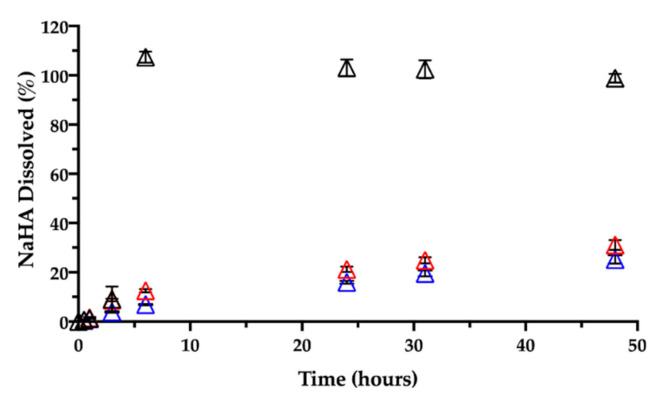
Release of sodium hyaluronate in 0.9% NaCl at 37 °C from films aged: 1 day (black), 3 months (red) and 7 months (blue). Data are expressed as mean ± SD (*n* = 3).

Together with CisPt, the NaHA dissolved from the film was quantified during the same release rate test (Figure 10).

In the early times, i.e., within the first 3 h, there was almost 10% (9.2 ± 5.2%) of NaHA in solution, reflecting the system’s initial swelling in saline medium. An hour later, no film was visible in the vial due to its complete dissolution, in line with 100% NaHA quantified in the release medium at 6 h. As previously observed, based on CisPt/NaHA complex formation, CisPt crosslinked the polymer. This made the film not immediately soluble in saline, whereas the placebo film dissolved in saline as rapidly as in water. It is worth noticing that, for the drug-loaded film, its full dissolution in saline occurred in correspondence of about 50% of drug released. This suggests that, after the film was gone, drug release rate was still controlled by the coordination equilibrium between platinum and hyaluronan. This is relevant thinking back to the film effect observed in vivo and consequent drug bioavailability.

Then, a dedicated experiment was carried out in the same conditions (medium, temperature) to see the influence of the film age on CisPt release. This stability-related investigation tested films that were 3 and 7 months old. Figure 9 and Figure 10, respectively, show CisPt release and NaHA dissolution for the aged films in comparison with the freshly manufactured system.

Apparently, there was no significant difference in the release of CisPt depending on the film age, as the dissolution profiles were practically superimposed. In all cases, a minimum of 88% of CisPt was released in 48 h. Conversely, both older films behaved very differently from the fresh film with respect to hyaluronan dissolution, as they continued to release NaHA rather slowly. There was no significant difference in the profiles of NaHA dissolved between the 3-month-old and the 7-month-old films (*p* > 0.05). Macroscopically, both these films rolled-up at the beginning of the test to form the typical cylinder that was still there after 48 h. The striking difference was between them and the 1-day-old film as the latter disappeared within the sixth hour, leaving 100% NaHA in solution.

In summary, the storage time after film manufacturing did not lead to differences in terms of CisPt release but did affect the film “behavior” in terms of polymeric matrix dissolution. It seems that aging somehow made film dissolution less dependent on drug release. In fact, the fresh film’s dissolution was governed by platinum complexation and crosslinking action, and the film polymer network was dismantled in correspondence of 50% of drug released. In contrast, even after 90% of drug released from the older films, the swelling and dissolution of the NaHA polymer were slow, as if something else other than platinum now crosslinked the polymer chains. We made the hypothesis that during storage, a chemical modification occurred to hyaluronan leading to the formation of an auto-crosslinked polymer network. In this regard, the hyaluronan polysaccharide can form intra- and inter-molecular esters based on the reaction between the carboxyl group and hydroxyl groups on the same and/or different polymer chain [41]. The resulting auto-crosslinked polymer has different rheological properties and sensitivity to enzymatic cleavage by hyaluronidase [42].

In all previous experiments we never found 100% of CisPt released (on dry basis). Indeed, platinum quantification in the film confirmed that the actual drug content complied with the theoretical concentration (1% *w*/*w*). Hence, we tested whether complete release could be achieved increasing the concentration of chloride ions in the dissolution medium. We used for this purpose a 1.8% (*w*/*v*) NaCl solution (hypertonic). There was no difference in CisPt release nor in NaHA dissolution in the 48 h experiment (data not shown). We concluded that the missing drug remained associated with the film, having reached a concentration equilibrium with the dissolution medium. At 48 h the film endured perfectly rolled-up under the effect of age. The cylindrical geometry was lost at much longer times, namely beyond 4 days, when the system appeared deformed and highly hydrated. Still, a shapeless gel mass was present. Understanding of the film modification during storage is the object of an ongoing investigation.

## 4. Conclusions

The data obtained confirmed the hypothesis of the presence in the intracavitary film of a cisplatin/hyaluronan complex. The complex formed early, since the preparation of the film-forming mixture. In this mixture more than 90% of the anticancer drug was involved in the complex.

Cisplatin release from the film was limited in water, where the released drug quickly underwent hydrolysis. Conversely, release was substantially different in saline medium, where cisplatin was almost completely released from the complex under the effect of chloride ions. We discovered that drug release was not dependent on film complete dissolution. In fact, in saline dissolution medium, part of cisplatin was let go in the form of a complex with hyaluronan as the film dissolved; final drug liberation from the complex occurred when the carboxylate was replaced by the chloride ligand in coordinating platinum. This has to be similar to the in vivo situation, where cisplatin was circulating as a complex from which the free drug was obtained by the intervention of chloride ions.

Film dissolution in saline was significantly delayed as the film aged. Nevertheless, the older, less soluble films had no effect on CisPt release rate. This opens up the possibility that a different chemical crosslink, independent of the coordinated platinum, occurs between the hyaluronan chains.

The investigation of film dissolution rate, in addition to showing the drug release kinetics in vitro, contributed to the understanding of the role of the complex in bioavailability of cisplatin from the implanted film.

The analytical determination of free cisplatin and CisPt/NaHA complex in the dissolution medium, described in this work, responds to the EMA request specified in the Protocol Assistance letter, i.e., the accompanying document for the pharmaceutical, preclinical and clinical development for the preparation of CTD dossier to submit for the authorization to market of the cisplatin film. What remains to validate is the analytical method for in vivo pharmacokinetics.

## Supplementary Materials

The following are available online at https://www.mdpi.com/1999-4923/13/3/362/s1, Figure S1: SEC-HPLC chromatogram of PVA solution (0.40 mg/mL) in water.
