# Supplementary material for: Orphan Designation and Cisplatin/Hyaluronan Complex in an Intracavitary Film for Malignant Mesothelioma
Source: Pharmaceutics. 2021 Mar 9;13(3):362. doi: 10.3390/pharmaceutics13030362 (PMC8000699; doi:10.3390/pharmaceutics13030362)
Supplement: Supplementary file 1 [file pharmaceutics-13-00362-s001.pdf]

pharmaceutics

Supplementary Materials: Orphan Designation and Cisplatin/Hyaluronan Complex in an Intracavitary Film for Malignant Mesothelioma

Sabrina Banella 1,†, Eride Quarta 2,†, Paolo Colombo 3, Fabio Sonvico 2, Antonella Pagnoni 4, Fabrizio Bortolotti 1 and Gaia Colombo 1,*

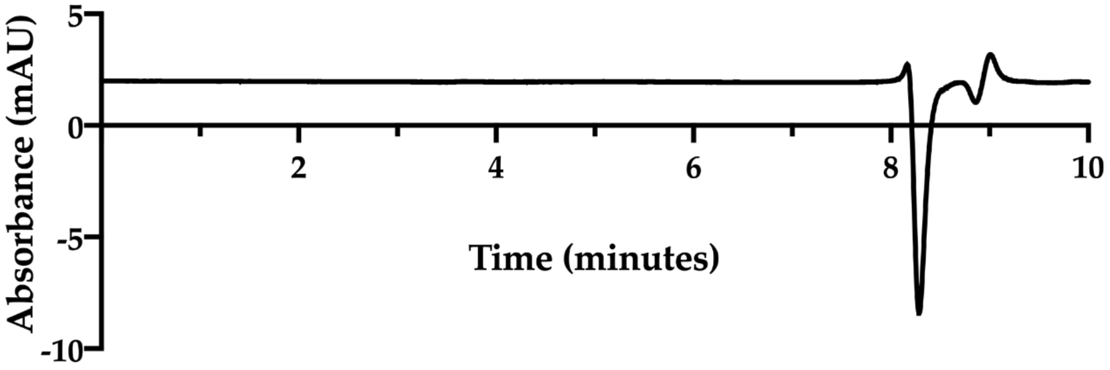

Figure S1. SEC-HPLC chromatogram of PVA solution (0.40 mg/mL) in water.

Citation: Banella, S.; Quarta, E.; Colombo, P.; Sonvico, F.; Pagnoni, A.; Bortolotti, F.; Colombo, G. Orphan Designation and Cisplatin/Hyaluronan Complex in an Intracavitary Film for Malignant Mesothelioma. *Pharmaceutics* **2021**, *13*, 362. https://doi.org/10.3390/pharmaceutics13030362

Received: 19 February 2021
Accepted: 4 March 2021
Published: 9 March 2021

Publisher's Note: MDPI stays neutral with regard to jurisdictional claims in published maps and institutional affiliations.

Copyright: © 2021 by the authors. Licensee MDPI, Basel, Switzerland. This article is an open access article distributed under the terms and conditions of the Creative Commons Attribution (CC BY) license (http://creativecommons.org/licenses/by/4.0/).
